# Viability Costs of Reproduction and Behavioral Compensation in Western Mosquitofish (*Gambusia affinis*)

**DOI:** 10.1371/journal.pone.0110524

**Published:** 2014-11-03

**Authors:** Clinton T. Laidlaw, Jacob M. Condon, Mark C. Belk

**Affiliations:** Department of Biology, Brigham Young University, Provo, Utah, United States of America; CNRS, France

## Abstract

The cost of reproduction hypothesis suggests that current reproduction has inherent tradeoffs with future reproduction. These tradeoffs can be both in the form of energy allocated to current offspring as opposed to somatic maintenance and future reproduction (allocation costs), or as an increase in mortality as a result of morphological or physiological changes related to reproduction (viability costs). Individuals may be able to decrease viability costs by altering behavior. Female western mosquitofish, *Gambusia affinis* experience a reduction in swimming ability as a consequence of pregnancy. We test for a viability cost of reproduction, and for behavioral compensation in pregnant female *G. affinis* by measuring survival of females in early and later stages of pregnancy when exposed to predation. Late-stage pregnant females experience a 70% greater probability of mortality compared to early-stage pregnant females. The presence of a refuge roughly doubled the odds of survival of both early and late-stage pregnant females. However, there was no interaction between refuge availability and stage of pregnancy. These data do not provide evidence for behavioral compensation by female *G. affinis* for elevated viability costs incurred during later stages of pregnancy. Behavioral compensation may be constrained by other aspects of the cost of reproduction.

## Introduction

Organisms incur costs to future reproduction as a consequence of current reproduction. The relative amount of energy and time that an organism invests in current reproduction is reflected as a cost to the potential for future reproduction (i.e., cost of reproduction hypothesis; [1], [2]. Costs to future reproduction can be categorized as allocation costs or viability costs. Allocation costs arise because resources allocated to current reproduction cannot be used for future reproduction [1]. Viability costs arise from physiological or morphological changes that result from current reproduction that lead to increased mortality rates compared to non-reproductive individuals [3] Viability costs can result from decreased physiological performance as a consequence of depletion of energy stores (i.e., physiological cost hypothesis; [4], [5]), or from additional morphological changes associated with producing and carrying eggs or developing embryos that produce a decrease in the motility of individuals or their ability to avoid predation (i.e., physical burden hypothesis; [6]; [7]). Some of the most apparent and well-studied consequences of reproduction are those associated with reduced mobility and escape velocity in pregnant or gravid females. Such reduction in mobility has been observed across many taxa; for example, in butterflies [8], fish [7], [9], [10], lizards [11], [12], snakes [5], birds [13], [14], and dolphins [15]. Although declines in performance arising from the physical burden of reproduction have been well documented, few studies have quantified actual cost to survival.

In fishes swimming performance is a major determinant of survival probability [16]. Swimming ability (i.e., steady or unsteady swimming measures) is strongly related to body form [16]–[19]. Livebearing fishes show a change in body form and increased overall mass in the latter stages of pregnancy. For example, in the livebearing fish *Brachyrhaphis rhabdophora*, females exhibit increased abdominal distension as pregnancy progresses [20], [21]. Pregnancy-related reduction in escape velocity has been observed in western mosquitofish (*Gambusia affinis*) [22], [23] and these studies have suggested that females experience a viability cost of reproduction as a consequence of the physical burden of livebearing. The argument is that changes in shape lead to reduced swimming ability which leads to increased mortality of pregnant females. However, the magnitude of this viability cost of reproduction associated with pregnancy or gravidity has rarely been quantified in vertebrates [24].

In response to an increase in predation risk, many organisms modify their behavior to reduce the probability of mortality. The threat-sensitivity hypothesis predicts that prey increase anti-predator behavior as risk of mortality increases [25], [26]. Many species avoid risky habitats, increase refuge use, or stay closer to refuge habitat when predators are near [10], [27]–[29]. Behavioral change in the frequency of use of refuges during times of increased predation risk should result in increased survival probabilities [30]. Organisms that effectively reduce viability costs can potentially gain higher lifetime fecundity and increase overall fitness. Selection should favor behavioral compensation to reduce viability costs of reproduction as long as the benefit to fitness (such as additional reproductive opportunities) outweighs the cost (reduced opportunity for foraging or other activities, i.e., [31]). Livebearing fishes exhibit antipredator behaviors including use of refuge habitats [32], [33]. However, how refuge availability influences viability costs of reproduction is not well known.

Previous work on livebearing fishes suggests that reduced locomotory performance associated with pregnancy is likely to result in increased mortality in pregnant females especially at later stages of pregnancy [18], [34]–[36], and that pregnant females should compensate for this increased probability of mortality by increasing the use of refuge habitats at later stages of pregnancy [27], [37]. However, we found no published studies that quantified viability costs of reproduction and refuge effects as a consequence of pregnancy. In this study, we test for viability costs of reproduction in female western mosquitofish (*Gambusia affinis*) by comparing mortality rates of early and late-term pregnant females to see if risk of predation increases with increasing volume during pregnancy. Additionally, we test whether pregnant females will use refuges with increased frequency as a means of behaviorally compensating for reduced escape velocity and increased susceptibility to predation.

## Methods

All animal work was done with the approval and supervision of the Institutional Animal Care and Use Committee at Brigham Young University. No regulations were broken. Permits were obtained from the Utah Division of Wildlife Resources for all bass and mosquitofish used in this experiment. Smallmouth bass were obtained via angling and all fish were sacrificed via overdoes of MS222 according to the direction of the Institutional Animal Care and Use Committee at Brigham Young University.

### Study System

Western mosquitofish (*Gambusia affinis*) is native to the southeastern US, but it has been introduced globally in temperate and tropical systems as a means of mosquito control [38]. Western mosquitofish are viviparous, producing broods that can range from 5 to over 100 offspring [39]–[41]. Gestation lasts about 22 to 25 days [39], [42] and as offspring develop they increase in volume resulting in an enlarged and extended abdominal area in females at later stages of pregnancy [23] and similar to many species of poeciliids, e.g., *Brachyrhaphis rhabdophora* [20], [21], and reduced swimming performance [22], [23]. Western mosquitofish are frequent prey of larger piscivorous fish (including small-mouth bass, *Micropterus dolomieui*) in their native habitat as well as many introduced systems.

Western mosquitofish for our experiments, were obtained from the Davis County Mosquito Abatement Program, Ogden Utah, USA. Reproductive females from the fish obtained ranged in size from 30–45 mm total length (TL). For our experiment we wanted to avoid confounding differences in length with differences due to stage of pregnancy, so we selected a more restricted size range of 35–40 mm TL. Western mosquitofish were maintained in large holding tanks (1100 liters), and fed a diet of enriched flake food when not being used in the experimental trials. Smallmouth bass were used as predators. Eight smallmouth bass of about equal length (205–256 mm SL) were collected from Jordanelle Reservoir, Summit County, Utah, and were maintained in holding tanks (1100 liters) covered with a shade-cloth to control temperature and prevent escape. Smallmouth bass were fed several western mosquitofish daily prior to and after their use in the experimental trials. Bass were not fed 24 hours prior to use in an experimental trial.

Experimental trials were conducted in tanks of the same size and manufacture as the holding tanks (i.e., 0.5 m water depth, 2.3 m diameter, 1100 liter volume, gray plastic, Rubbermaid stock tanks). Tanks were arranged in an outside, partially shaded area, and water temperature in the experimental and holding tanks varied between 15° and 20°C over the course of the day and night. We used aged tap water to fill the tanks. Tanks were arranged in an array separated by one meter to allow for ease of transfer and maintenance of experimental individuals and to maintain homogenous conditions among tanks. All tanks were continuously aerated, and maintained under a shade-cloth tent to minimize temperature fluctuation.

### Experimental Protocol

Our goal was to compare mortality rates between groups of females at different stages of pregnancy. Stage of pregnancy can only be exactly determined by dissection and staging of embryos by direct inspection. Obviously we could not dissect females prior to or after the experiments, so we used the mass-to-length ratio as a surrogate for stage of pregnancy. For a given length, low-mass individuals generally represent females in the early stages of pregnancy and high-mass individuals represent the latter stages of pregnancy [22]. To estimate the reproductive stage of live females we randomly selected 118 female western mosquitofish (*G. affinis*), measured TL (in mm) and mass (in mg), and used a linear regression of natural log-transformed mass and natural log-transformed length. Residuals from the mass-to-length relationship were used to assign females as either early- or late-stage pregnant. Individuals with a residual greater than 0.075 were considered to be late-stage pregnant (high volume to length ratio), individuals with a residual less than −0.075 were considered to be early-stage pregnant (low volume to length ratio), and all individuals with intermediate residuals (−0.075 to 0.075) were excluded from the experiment to ensure differentiation between groups.

To test for viability costs and behavioral compensation through increased refuge use in early and late-stage pregnant females, we allowed females in experimental tanks to be preyed upon by smallmouth bass for a limited time and then quantified survival. Early and late-stage females were randomly assigned to one of two refuge treatments in a fully crossed factorial design consisting of stage of pregnancy crossed with availability of a refuge as follows: early-stage females without a refuge, early-stage females with a refuge, late-stage females without a refuge and late-stage females with a refuge. To test for possible mortality resulting from handling stress (as opposed to predation), ten additional trials involving ten fish per trial (of both early- and late-stage pregnant groups) were run without a predator present. No mortalities were observed in these control trials, indicating that handling was not a significant source of mortality in experimental trials.

Treatments involving a refuge were utilized to determine to what degree refuge use as a predator avoidance behavior would increase survival in both early- and late-stage pregnant females, and to indicate whether late-stage females are able compensate for increased risk of mortality by increased use of refuges. Refuges were constructed from a plastic mesh (2 cm gap size) bent into a cylindrical shape measuring approximately.75 meters in diameter. Green, polypropylene rope was threaded through the bottom of mesh and allowed to float upward to simulate plant cover within the refuge. Refuges were then submerged in the center of the tank. The mesh size of the refuge did not allow smallmouth bass to access western mosquitofish inside the refuge.

To begin experimental trials: female western mosquitofish were selected from the stock population and measured, and their mass-to-length ratio was used to assign them to early-stage or late-stage pregnant groups. Females were tested in groups of ten individuals of their same stage of pregnancy. Groups were then randomly assigned to a treatment tank (with or without a refuge) resulting in four different treatments (early-stage without refuge, early-stage with refuge, late-stage without refuge and late-stage with refuge). Each block contained one or two of each treatment. A total of six blocks were run sequentially through time producing a total of nine replicates of each treatment. One replicate of each of two treatments was lost, resulting in eight successful replicates for both the early and late-stage with refuge treatments. The total number of mosquitofish used was 340.

Western mosquitofish were allowed to acclimate to conditions in the experimental tanks for one hour. To avoid specific predator effects, smallmouth bass were randomly assigned to tanks within each block and subsequently introduced to the tanks one hour after the mosquitofish. Flake food was placed along the perimeters of the tank ensuring that individuals would need to leave the refuge to feed. Tanks were then covered with a 2.5 cm mesh material to prevent the bass from escaping. One or two replicates of each of the four treatments were run concurrently depending on availability of female western mosquitofish and smallmouth bass.

To determine the effect of stage of pregnancy and the presence of a refuge on survival, trials were run for times varying from 6 to 24 hours. The run time for each trial was dependent on the time required for 40–60% of western mosquitofish from any one of the experimental tanks to be consumed (based on a visual assessment). Once such mortality was observed in a single tank, all concurrent trials were stopped, the predators were removed, and surviving western mosquitofish and predators were returned to separate recovery tanks. Trials varied in duration because it was necessary that no trial concluded without mortality or after complete mortality had occurred (as no further mortality would be possible). Differences in the lengths of trials were attributable to variation in predator behavior. Surviving western mosquitofish that were removed at the end of the trial were not reused in subsequent trials. Trials were run from late June to early September, 2004.

### Measurement and Statistical Analysis

Following each trial, fish were counted and the number surviving in each tank was determined. We used logistic regression to test for the effect of stage of pregnancy and availability of a refuge on the probability of survival (i.e., viability costs of reproduction). The response variable was the number surviving compared to the total number that began the trial (i.e., 10 individuals per trial), and the independent variables were stage of pregnancy, and refuge availability. We included the interaction between stage of pregnancy and refuge availability to test for behavioral compensation of late-stage pregnant females. Significance was determined at p<0.05. Analysis was done using the Logistic procedure in SAS version 9.3 (SAS Institute, Cary, North Carolina, USA).

## Results

**Figure 1:**
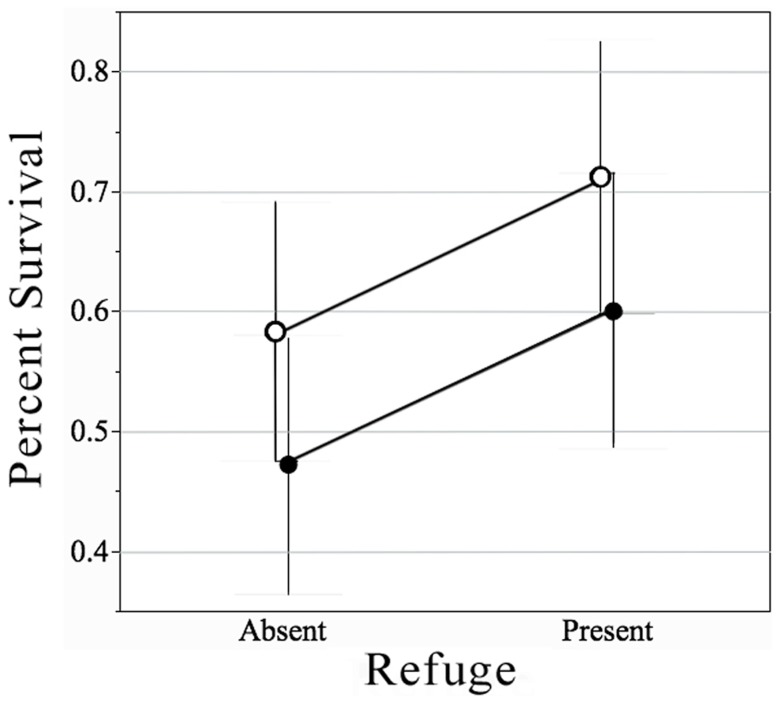
Mean (± SE) percent survival of female western mosquitofish in the presence of a predator based on stage of pregnancy and availability of refuge. Open circles represent early-stage pregnant females and closed circles represent late-stage pregnant females.

Females in the later stages of pregnancy experienced decreased probability of survival compared to early-stage pregnant females, and all females showed increased survival in tanks with a refuge present. The interaction between the stage of pregnancy and refuge availability was not significant (Table 1). Females in later stages of pregnancy had about a 33% higher relative risk of mortality compared to individuals in early stages of pregnancy (odds ratio  = 1.699, 95% confidence limits 1.07–2.71). The presence of a refuge decreased the relative risk by about 27% (odds ratio  = 0.55, 95% confidence limits 0.34–0.87) for early- and late-stage pregnant females combined (Fig. 1). Although there is no statistical interaction between stage of pregnancy and refuge availability, the effect size (measured as relative risk) of refuge availability is somewhat different between earl- and late-stage females. The odds-ratio is higher between early- and late-stage pregnant females with refuge compared to those without, resulting in a difference in relative risk of 16.6% in early- and late-stage pregnant females with and without refuges available (Table 2).

**Table 1:** Type 3 tests of effects of predictor variables from logistic regression analysis of probability of survival of female western mosquitofish in the presence of a predator.

Effect	Degrees of freedom	Wald Chi-Square	P
block	5	29.3525	<.0001
stage of pregnancy	1	4.9583	0.0260
refuge	1	6.3577	0.0117
stage of pregnancy by refuge	1	0.0224	0.8810

**Table 2:** Survival percentages and marginal odds ratios for female western mosquitofish at early and late stages of pregnancy and with and without a refuge present.

	Early-Stage	Late-Stage	Marginal Odds Ratio
Refuge Absent	58.9	47.8	1.646
Refuge Present	73.8	62.5	1.768
Marginal Odds Ratio	.526	.565	

## Discussion and Conclusions

Morphological or physiological effects associated with the latter stages of pregnancy result in dramatically increased mortality due to predation in western mosquitofish. High-volume to length ratio associated with later stages of pregnancy results in decreased escape velocity in *G. affinis* [22], and *Poecilia reticulata* (Trinidadian guppy) [7]. The correlation between reproduction and decreased locomotory performance has also been demonstrated among reptiles [11], [24], [43]–[47], birds [13], [14] and in other species of fish [9], [10]. These previous studies suggest an effect of the physical burden of pregnancy or egg production on swimming performance (i.e., escape velocity), and provide support for the physical burden hypothesis as a mechanism for the viability cost of reproduction documented here. We cannot rule out the physiological cost hypothesis, and of course, both effects could occur simultaneously. To our knowledge this is the first experimentally quantified demonstration of viability costs from reproduction as a consequence of predation. Such costs may be common among taxa that demonstrate a morphological change associated with production of eggs or pregnancy.

The presence of a refuge led to increased survival. The refuge effect is well documented in predator-prey systems such as in creek chub (*Semotilus atromaculatus*) [29], and Trinidad guppies (*Poecilia reticulata*) [48]. Female western mosquitofish in the no-refuge treatments typically congregated along the edge of the tank; whereas, females in the refuge treatments were often found in the refuge, and they moved in and out of the refuge in response to the behavior and position of the predator. Females at all stages of pregnancy appeared to recognize and use the refuge.

Although there was a clear effect of the refuge, there is no significant statistical interaction in survival percentage between stage of pregnancy and refuge availability. However, the relative risk of late-stage pregnant females compared to early-stage pregnant females (i.e., the pregnancy effect) is greater when a refuge is present (relative risk  = 1.27 without refuge, and 1.43 with refuge). Although this represents a small difference when viewed as absolute percent survival, when we consider the change in relative risk it becomes a nontrivial difference of 16.6%. In other words, the change in absolute percent survival between refuge and no-refuge treatments, has a differential effect size (calculated as relative risk) on early-stage and late-stage pregnant females because late-stage pregnant females have an overall lower survival percentage. Several studies have shown that the presence of a predator is related to behavioral compensation by prey [26], [34], [49]–[51]. Presumably, female western mosquitofish could modify their behavior to compensate for the reduction in predator escape ability resulting from pregnancy, but they experience higher relative mortality when refuges are available.

Why do late-stage pregnant females not exhibit increased refuge use as a means of behavioral compensation for reduced escape probability? We explore two possible explanations for the lack of behavioral compensation. First, females may be constrained by the lack of resources available in refuge environments. Reduced resource acquisition would leave less energy available for key life-history components (growth, somatic maintenance, future if not current reproduction). As an iteroparous organism reproduction is not limited to a single bout and any period of reduced foraging may delay, reduce, or eliminate opportunities for future reproduction (ie. yolking of eggs, attaining larger body size, survival). There is also evidence for slight matrotrophy in western mosquitofish [52], which would indicate that reduced foraging time may incur a cost to current offspring as well as future offspring. Females may abstain from feeding during the final stages of pregnancy due to spatial constraints [53] in which case there may be increased need for foraging preceding fasting to insure survival. In environments where refuge use comes at a cost to resource acquisition and refuge use decreases mortality, behaviors will be favored that minimize the ratio of mortality to resource gain [29]. In western mosquitofish these cost in terms of reduced foraging opportunity may outweigh the benefit of increased refuge use by late-stage pregnant females.

Second, the western mosquitofish used in this experiment were obtained from captive populations. Because predation has not been present in this population for many generations, selection likely favors behaviors related to resource acquisition to a greater degree than in populations occurring with predators present. The effects of population density may increase further the strength of selection to increase foraging behaviors as competition is a selective force that generally favors larger offspring and behavior that facilitates growth of embryos [54]. Rapid evolution of behavior and morphology can occur in live-bearing fish in less than 20 years as a result of the removal of predators from an environment [55]. Thus, selection in hatchery populations may not favor an increase in refuge use behaviors and may in fact favor decreased use of refuges across reproductive stages. It would be of interest to compare refuge use behaviors in western mosquitofish from high predation and low predation environments.

There is a viability cost due to pregnancy in western mosquitofish, which we have observed in the form of actual mortality due to predation. The likelihood of survival from predation decreases as volume increases. The use of refuges serves to decrease the probability of mortality. Increased mortality in late-stage pregnant females is not counteracted by an increase in refuge use. Thus, western mosquitofish, like many other species that incur a reduction in performance associated with pregnancy, must balance behaviors that reduce the odds of mortality from predation and behaviors that permit the acquisition of resources requisite for survival and reproduction.
